# Mental health consequences for adolescents during the Russian invasion of Ukraine: protocol for the Ukraine Adolescent Mental Health Study

**DOI:** 10.3389/frcha.2025.1637011

**Published:** 2025-09-01

**Authors:** Olga Osokina, Sanju Silwal, Minja Westerlund, Emmi Heinonen, Susanna Hinkka-Yli-Salomäki, Gennadiy Putyatin, Yuliia Yaschchyshyna, Norbert Skokauskas, Matthew Hodes, Andre Sourander

**Affiliations:** ^1^ Department of Psychiatry, Psychotherapy, Addiction Psychiatry and Medical Psychology, Donetsk National Medical University, Kropyvnytskyi, Ukraine; ^2^ Research Centre for Child Psychiatry, INVEST Flagship, University of Turku, Turku, Finland; ^3^ Regional Centre for Child and Youth Mental Health and Child Welfare, IPH, Norwegian University of Science and Technology, Trondheim, Norway; ^4^ Division of Psychiatry, Imperial College London, London, United Kingdom; ^5^ Department of Child Psychiatry, Turku University Hospital, Turku, Finland

**Keywords:** war, adolescents, mental health, protocol, Ukraine

## Abstract

**Background:**

In February 2022, Russia launched a full scale-invasion of Ukraine, which is the largest European ground offensive since the Second World War. However, the Russian-Ukrainian war began in 2014, when Russia invaded and annexed the Crimea peninsula and occupied part of the eastern regions of Ukraine. This prolonged exposure to war, with its many casualties and massive displacement, has negatively affected the mental health of adolescents, although a comparison of the impacts on adolescents exposed to the various stages of war has not been documented. Our aim is to explore the effects of differential wartime traumatic stressor exposure and displacement on the mental health of adolescents exposed to the Russian invasion in Ukraine since 2014.

**Methods:**

The Ukraine Adolescent Mental Health Study (UAMS) is a time-trend study comprising two cross-sectional school surveys. The first survey was carried out in 2016–2017, two years after eastern Ukraine was invaded by Russia. The second survey was conducted after the 2022 full-scale Russian invasion. Both surveys used the same method and included participants aged 11–17 years from two areas in Ukraine, the Donetsk region and the Kirovograd region. In 2016–2017, we focused on adolescents living in the eastern Donetsk region who had been exposed to war since 2014 and those living in the central Kirovograd region, which was not directly affected by the invasion. The new survey will enable us to compare exposure to traumatic wartime stressors and mental health problems among adolescents over time and between the two regions. Several standardized tools will be used to assess post-traumatic stress disorder, depression, anxiety, suicidal ideation, suicide attempts and self-harm behavior.

**Discussion:**

This study will provide a unique opportunity to examine the escalating psychological consequences of the ongoing war on adolescents in Ukraine. Such information is crucial for understanding adolescents’ mental health needs, and thus for providing psychosocial support and developing mental health interventions.

## Background

The war in Ukraine started in February 2014, when Russia annexed the Crimean Peninsula and occupied the eastern part of the country. Russia launched a full scale-invasion of Ukraine in February 2022 in what has become the largest European ground offensive since the Second World War. By mid-2023, at least 11,973 Ukrainian civilians had been killed, 3.7 million had been internally displaced and 6.7 million people had sought refuge in other countries (1, 2). The war has resulted in enormous suffering and has had extensive negative impacts on people lives and well-being (3).

Research indicates that the ongoing war has led to an increase in war-related traumatic stressors and psychological distress (4, 5). Most studies have focused on adults or refugees with elevated levels of depression, anxiety, post-traumatic stress disorder (PTSD) or sleep disturbances (4–17). Few studies have been conducted on children or adolescents who have lived in Ukraine since the full-scale invasion in 2022 (18–20). One study found that 12.6% of adolescents aged 7–17 had experienced post-traumatic stress disorder symptoms (18); and another study reported internalizing and externalizing problems in children with a mean age of 10 (19). A study conducted among adolescents who were 15 and older and living in Ukraine or abroad showed that those living abroad had an increased risk for mental health problems compared to those living in Ukraine (20). However, these studies had some limitations, as they were based on cross-sectional surveys and had a narrow range of outcome measures, e.g., they did not assess suicidal behavior. Obtaining reliable information on the effects of war requires repeated cross-sectional surveys using the same sampling approaches and measures. Another research approach is to compare regions affected by war to those that have been unaffected, can provide the conditions that are as close as possible to a natural experiment.

The present study, the Ukraine Adolescent Mental Health Study (UAMS), is unique as it includes two cross-sectional studies. One was conducted when Ukraine was first exposed to the local war in the eastern part of the country in 2014 and the other is being carried out after the full-scale war that started in 2022. In 2016–2017, we compared exposure to wartime traumatic stressors and the mental health of adolescents living in two regions: the Donetsk region of eastern Ukraine, which had experienced armed conflict since 2014, and the Kirovograd region, in central Ukraine, which had not been directly affected by war at that time. Based on the 2016–2017 data, we have previously reported that adolescents living in the local war-affected Donetsk region had higher levels of wartime traumatic stressors, psychopathology, suicidality and self-harm behavior than their peers in the Kirovograd region (21, 22). A similar cross-sectional study is ongoing among adolescents of the same age group from the same two regions using an identical design and the same methods. Collecting new data during the full-scale war provides us with a remarkable opportunity to compare the mental health of adolescents exposed to the various stages of war. Our aim is to explore the effects of differential wartime traumatic stressor exposure and displacement on the mental health of adolescents exposed to Russian invasion since 2014. Four samples will be examined. The two samples from 2016 to 2017 will be used to examine mental health of adolescents living in the war-affected Donetsk region and the non-war-affected Kirovograd region. The new survey samples will be used to examine adolescents exposed to the full-scale war in Ukraine, who were originally from the local war-affected Donetsk region and the central Kirovograd region that was not directly affected by the 2014 local war. Adolescents who have experienced the full-scale war, and are originally from the local war Donetsk region, will have been exposed to war for a longer period than the other three groups. Therefore, we hypothesize that they will have experienced heightened levels of psychopathology, suicidality and wartime traumatic stressor exposures than the other groups.

## Methods

### Overall study design

**Figure 1:**
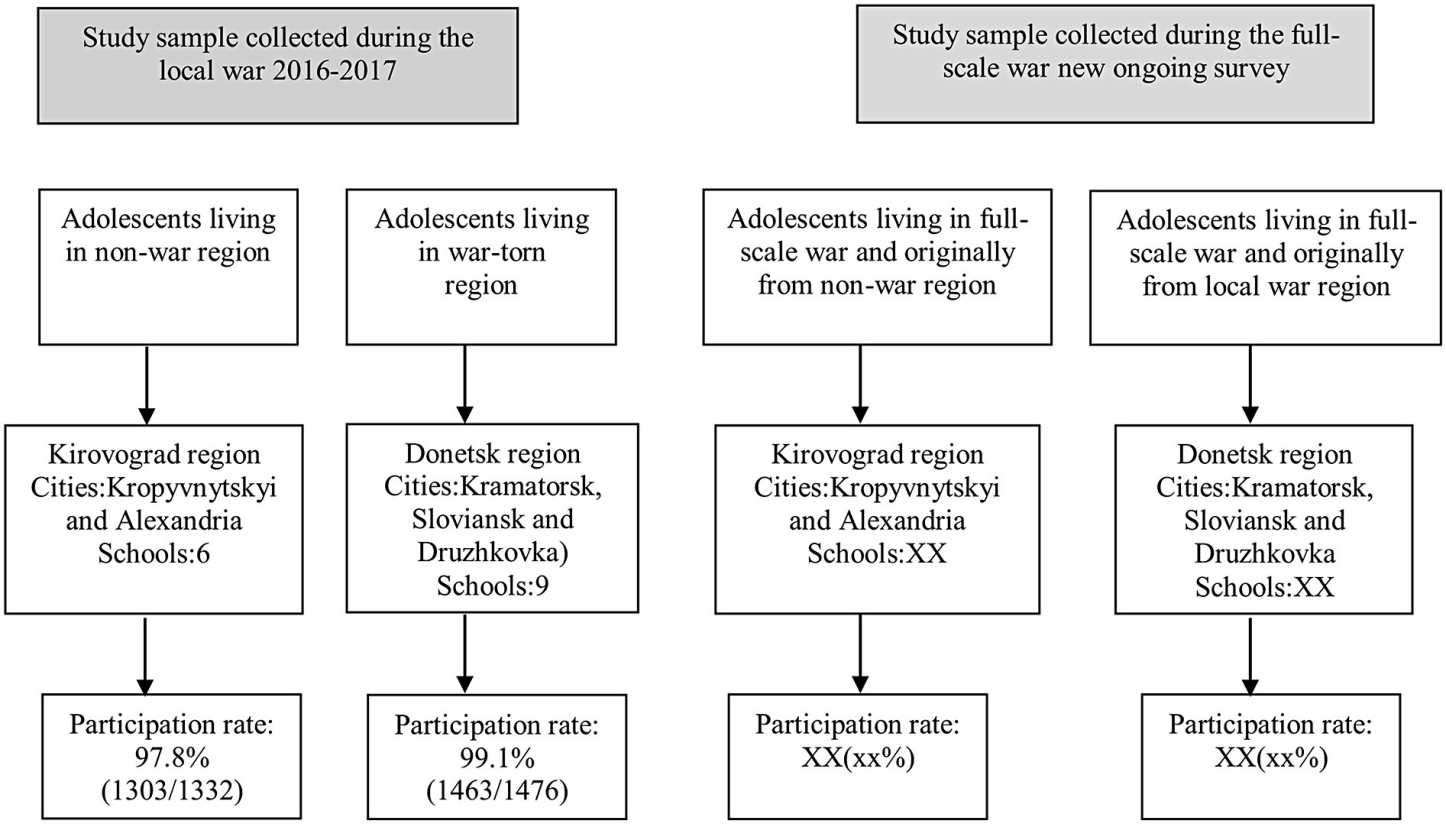
Flow chart of study design for the 2016–2017 and the new, ongoing survey.

The UAMS is a time-trend study, using school-based surveys carried out in 2016–2017 and an ongoing survey, currently in progress. Figure 1 illustrates the flowchart of the study design. The initial cross-sectional study was conducted from September 2016 to January 2017, more than two years after Russia invaded areas of eastern Ukraine in 2014. Participants were adolescents 11–17 years old, enrolled in grades 6–9, from two regions exposed to the war to different degrees. The Donetsk region, located in eastern Ukraine, has been affected by the war since 2014. Data were collected from three cities in this region: Kramatorsk, Sloviansk and Druzhkovka. The Kirovograd region, situated in central Ukraine, was a peaceful region in 2016–2017 and was not directly affected by the war. Data from the Kirovograd region were collected from two cities: Kropyvnytskyi and Alexandria.

Convenience sampling was used to select public schools in the two regions. Schools were contacted to confirm their willingness to participate in the study, and 15 schools were enrolled in 2016–2017: nine from the Donetsk region and six from the Kirovograd region. The purpose of the study and the nature of participation were explained to the teachers, who facilitated the distribution of the study details and informed consent forms. Only adolescents with parental or guardian consent were eligible to participate. On the day of the data collection, participants completed a paper-based questionnaire in Ukrainian or Russian in their classrooms, supervised by researchers and teachers. The questionnaire took approximately 45 min to complete. Response rates were 99.1% in the Donetsk region and 97.8% in the Kirovograd region, resulting in a final sample of 2,766 participants. There were only 42 non-participants, due to absence from school (11 in the Donetsk region, 26 in the Kirovograd region) or lack of parental consent (2 in the Donetsk region, 3 in the Kirovograd region).

We are currently conducting a repeated cross-sectional survey in the same two regions, targeting public schools in the same cities of the Donetsk and Kirovograd regions. We aim to enroll the same number of schools as with the 2016–2017 survey. Due to the ongoing war, many adolescents have been displaced to regions of Ukraine less affected by the war or they have left the country. Despite their change in residence, these adolescents continue to attend their original schools virtually. In this study, only adolescents residing in Ukraine are included.

Due to martial law in Ukraine and the Decree of the President of Ukraine, schools in unsafe regions have shifted from contact teaching to distance or mixed learning. Depending on the level of threat and proximity to the active combat zone, all educational institutions in eastern Ukraine (Donetsk region), have fully transitioned to remote learning. Meanwhile, in central Ukraine (Kirovograd region), schools have continued either in-person or in a mixed format (a combination of in-person and remote learning), depending on the level of immediate risk on a given day. Decisions regarding the mode of education have been made by school administrations based on official notifications from regional military administrations and the national civil defense system. The alert system includes both traditional means (sirens, radio signals) and modern digital tools, including mobile applications (such as “Air Alert” and various Telegram bots) synchronized with an automated threat monitoring system. This has enabled schools to respond promptly to missile, artillery or drone attacks and to adapt the educational process flexibly in accordance with the current security situation. Thus, the participants have had the opportunity to complete the questionnaire either in paper form or online using a using Google form. Those using the paper version complete the questionnaire in their classroom, while the Google forms are completed using the participants' personal digital devices or devices provided by the researchers. Using an online survey method has enabled the inclusion of schools conducting virtual classes, which has facilitated data collection from Donetsk region, something that would have been impossible otherwise. The paper questionnaire takes approximately 45 min to complete, while the online version takes about 30 min. We anticipate including around 3,000 participants in the current survey. Participants will have access to psychological consultation provided by trained mental health professionals to support their mental health when needed.

### Measures

A full list of the measures used in the UAMS study is shown in Table 1. The demographic information includes sex (girl or boy), age, region of residence, parental employment status and family structure. A number of questions have been added to the new survey to capture information related to displacement after the Russian full-scale invasion in 2022. The new questions include place of residence before the onset of the full-scale war, current place of residence, duration of time spent away from the native home and the frequency of relocation.

**Table 1:** Measures used in the UAMS study.

	Mental health outcomes	Instruments	Number of items	Categorization (score)
1	Wartime traumatic stressor exposure		18	No, yes
2	Post-traumatic stress disorder	Harvard Trauma Questionnaire (23)	16	No PTSD (<2.5), probable PTSD (≥2.5)
3	Anxiety	Generalized Anxiety Disorder-7 (28)	7	None (0–4), mild (5–9), moderate (10–14), severe (15–21)
4	Depression	Patient Health Questionnaire-9 (27)	9	None (0–4), mild (5–9), moderate (10–14), moderately severe (15–19), severe (20–27)
5	Suicide attempt, suicidal ideation, self-harm behavior		3	No, yes
6	School environment		4	Never, sometimes, often, always
7	Psychosomatic symptoms		3	Hardly ever, less frequently, at least once a month, at least once a week
8	Alcohol, cigarette and illegal drug use		3	Never, rarely, weekly, daily
9	Bullying		6	Not at all, less than once a week, more than once a week, most days
10	Friends		1	Lots of friends, some friends, no friends, virtual friends
11	Loneliness		1	Never, rarely, sometimes, most of the time, always
12	Help seeking		2	No, considered help, sought help
13	Coping	Coping Strategy Indicator (30)	33	Very low, low, medium, high, very high
14	Family functioning	General Functioning Scale of the McMaster Family Assessment Device (31)	12	Strongly disagree, disagree, agree, strongly agree

Exposure to wartime traumatic stressors is assessed using original questions developed by Ukrainian child and adolescent psychiatrists and psychologists working with adolescents exposed to war events. The questionnaire comprises 18 items addressing various types of war-related events, with yes/no responses. Adolescents in the new survey are asked to report wartime traumatic stressors that they have experienced since the beginning of the full-scale war in 2022. Five items have been added to the new survey to elucidate the nature of war exposure. PTSD symptoms are assessed with the Harvard Trauma Questionnaire (HTQ), an instrument used widely for adults and adolescents (23, 24). This self-report tool consists of the first 16 items of the PTSD criteria in the Diagnostic and Statistical Manual of Mental Disorders, Fourth Edition (DSM-IV). Respondents rate the frequency of symptoms experienced in the past six months on a scale from zero (not at all) to four (extremely frequent). Depressive symptoms are assessed using the nine-item Patient Health Questionnaire (PHQ-9), a self-administered tool that has been extensively used with both adults and adolescents (25–27). Anxiety symptoms are assessed with the Generalized Anxiety Disorder-7 (GAD-7) questionnaire, which has been widely used with both adults and adolescents (28, 29). This self-report tool comprises seven items based on the DSM-IV diagnostic criteria for generalized anxiety disorder. Respondents rate their symptoms in the last two weeks on a four-point Likert scale; zero for “not at all”, one for “several days”, two for “half of the days”, and three for “nearly every day”. The total score can range from 0 to 21.

This study also assesses suicidality and self-harm behavior. Adolescents are asked whether they have intentionally hurt themselves by cutting or burning their skin, if they have seriously thought about taking their own life or if they have attempted to take their own life. Bullying is assessed with four questions asking how often they have been bullied, or have bullied others, at school and away from school in the past six months. The new survey includes additional questions on cyberbullying. Psychosomatic symptoms are assessed using three questions. Adolescents are asked if they have experienced distracting headaches or recurring abdominal pain and if they have had problems falling asleep or sleeping. Questions on substance use include how often adolescents use alcohol, smoke cigarettes or other nicotine products, and whether they use or have ever tried any illegal drugs. The need for outside help is assessed using two questions asking adolescents if they feel they would require outside help with problems, feelings, behavior or emotional trouble. Adolescents' coping strategies are assessed using the Coping Strategy Indicator (CSI), which is a self-report measure of situational coping encompassing strategies of avoidance, problem solving and seeking social support (30). The new version of the survey includes questions related to friends, loneliness and family functioning. Adolescents are asked how many close friends they have and how often they have felt lonely in the last 12 months. Family functioning is assessed using the General Functioning Scale of the McMaster Family Assessment Device (FAD-GFS), which contains six items reflecting healthy family functioning and six items reflecting unhealthy functioning (31).

For the 2016–2017 survey, all measures were translated from English into Ukrainian and Russian and then back-translated into English to assess their accuracy. For the new version, the same process is being followed except the translations is being to Ukrainian only.

### Statistical analysis

A descriptive analysis will be performed to describe the adolescents' demographic characteristics. Means and standard deviations will be calculated for continuous variables and frequencies, and percentages will be calculated for categorical variables. We will use regressions models to explore risk and protective factors associated with outcomes including mental health problems, suicidality, substance use and bullying. Odds ratios together with 95% conﬁdence intervals will be used to estimate the strength of the associations. A test of interaction will be conducted between sex and city in association with different outcomes. Statistical significance will be based on a two-sided *p*-value of <0.05. All statistical analyses will be performed with SAS 9.4 software (SAS Institute, Cary, NC, USA).

## Discussion

The ongoing war in Ukraine is putting a substantial mental health burden on adolescents over an extended period of time. There is an urgent need for epidemiological studies to document and address their mental health needs. The UAMS study is a novel time-trend study designed to assess the impact of both the local and full-scale Russian invasion on adolescent mental health. This approach will reveal the mental health impacts of adolescents exposed to various stages of the war, capturing the effects of diverse wartime exposures and displacement. The strengths of the study include its large sample size and use of standardized tools that help assess the prevalence of mental health problems and identify associated risk and protective factors. The study also has limitations, for example its reliance on self-reports, although this method is commonly used in war situations as collecting cross-informant reports from parents and teachers is quite challenging in such contexts. We did not collect information on the pre-war mental health of participants as it may be sensitive. However, our study is population-based and regionally representative. We expect that their pre-war mental health problems levels reflect that of the general youth population (32–34). Additionally, we lack information on their general health status.

Even before the full-scale war, Ukrainian adolescents were facing large unmet mental health needs (35, 36). The escalation of the Russian invasion has further exacerbated this situation and put an additional strain on the existing child and adolescent mental health infrastructure and the capacities of the workforce (37). The findings of the study will be crucial to understanding the level of psychosocial distress experienced by adolescents, underscoring the urgency for acute interventions and rehabilitation. The study will identify risk and protective factors and generate further research questions about the interplay between these factors, influencing mental health problems and individual trajectories.

## Data Availability

The original contributions presented in the study are included in the article/Supplementary Material, further inquiries can be directed to the corresponding author.
